# The Impact of Age and Sex on Left Ventricular Function Based on Transthoracic Echocardiograms

**DOI:** 10.31083/RCM38779

**Published:** 2025-06-27

**Authors:** Lu Tang, Rui Wu, Chen Cheng, Zheng Li, Yang Hua, Jin-Yu Sun, Yan-Juan Zhang, Wei Sun, Xiang-Qing Kong

**Affiliations:** ^1^Department of Cardiology, The First Affiliated Hospital with Nanjing Medical University, 210029 Nanjing, Jiangsu, China

**Keywords:** transthoracic echocardiogram, left ventricular systolic function, left ventricular diastolic function, age, gender

## Abstract

**Background:**

This study aimed to reveal the age- and gender-related differences in left ventricular function among patients with normal cardiac structure.

**Methods:**

A retrospective analysis was performed on 10,853 individuals with normal cardiac structures undergoing transthoracic echocardiography (2017–2020). We performed distribution analysis using kernel density estimation with Gaussian kernels and created smooth trajectories based on generalized additive models. Moreover, correlation analysis and multivariable regression were applied to evaluate the impact of age and gender on ventricular function.

**Results:**

A weak but statistically significant correlation was found between age and ejection fraction (B-coefficient = –0.077, *p* < 0.001). Females presented with a higher early diastolic mitral inflow velocity (E)/ early diastolic mitral annular tissue velocity (e') ratio than males across all age decades (*p* < 0.001). However, age demonstrated stronger associations with functional parameters in individuals below 51.4 years (both genders, *p* < 0.001). Multivariable regression analysis indicated that age and the male gender were independent predictors of reduced septal and lateral e' velocities (both *p* < 0.001), with males showing lower values (septal B-coefficient = –0.290; lateral B-coefficient = –0.463).

**Conclusion:**

This study provided the distribution of left ventricular systolic/diastolic function across age decades in males and females and highlighted the clinical importance of monitoring ventricular function even for patients with normal cardiac structure.

## 1. Introduction

Left ventricular (LV) function has been demonstrated as an important diagnostic and prognostic factor of multiple cardiovascular diseases [1, 2, 3] and a wide range of other types of diseases [4]. Despite the advances in cardiac computed tomography (CT) and magnetic resonance imaging (MRI), transthoracic echocardiogram (TTE) is still the most widely used non-invasive method for evaluating LV function and morphology, owing to its unique advantages in providing real-time images of a beating heart [5].

Ejection fraction (EF) is a load-sensitive measure of systolic function, which is calculated based on the following formula: EF = (end-diastolic volume – end-systolic volume)/end-diastolic volume. Early diastolic mitral annular tissue velocity (e’) is acquired at the lateral and septal basal regions. Early diastolic mitral inflow velocity to early diastolic mitral annular tissue velocity (E/e’) ratio, a feasible and reproducible index, is often used to estimate LV filling pressure, while early to late diastolic transmitral flow velocity (E/A) reflects diastolic function. EF, E/A, and E/e’ ratio have been routinely used to diagnose disease, make therapeutic decisions [6], and predict prognoses [7, 8].

Accumulating studies have created a growing awareness of the age- and gender-specific differences in heart function and disease progression [9, 10]. Males and females have varied predispositions to LV dysfunction and manifest different clinical profiles [11, 12], and females generally tend to have a poorer prognosis than males [13]. The risk of cardiovascular diseases was also reported to rise in females at higher left ventricular ejection fraction (LVEF) than males [14]. Moreover, LV function and structure changes with advancing age in healthy individuals, including increased wall thickness, prolonged pre-ejection period, decreased shortening along the long axis and enhanced ventricular twist [9, 15, 16]. Due to the aging population worldwide, it becomes increasingly significant to distinguish normal age-related changes in LV function from pathological state. Accordingly, accurate evaluation and interpretation of LV parameters are fundamental for risk stratification and clinical decision making for cardiovascular diseases.

Despite the growing awareness of the impact of age and gender on LV systolic and diastolic function, the gender and age-specific values for LV function are still lacking. Currently only a few studies based on small populations with limited age ranges have reported the impact of age on LV function [17, 18, 19], and the results were generally inconsistent [18, 20, 21, 22]. This study aimed to provide the distribution of LV systolic/diastolic function across age decades in males and females using TTE and reveal the impact of age and gender on LV function, with a focus on the Chinese population.

## 2. Methods

### 2.1 Echocardiography

We retrospectively collected TTE data from the digital echocardiogram database, which stores all of the TTEs performed in the First Affiliated Hospital of Nanjing Medical University. TTEs were performed by commercially available echocardiographic instruments (Ge Vivid E9, Philips-iE33, Acuson Sc 2000 or EPIQ 7c, Amsterdam, the Netherlands). Measurements were routinely performed following standardized methodologies recommended by the American Society of Echocardiography [5]. LV ejection fraction was calculated based on Teichholz methods [23]. Early diastolic mitral inflow velocity (E) and late diastolic mitral inflow velocity (A) were assessed in the left lateral decubitus position at the mitral tip by pulsed Doppler echocardiography, whereas early diastolic mitral annular tissue velocity (e’) was measured at the lateral and septal position by tissue Doppler imaging. All the TTE data were analyzed by at least 2 doctors, and at least one senior cardiologist confirmed the results.

### 2.2 Data Collection

We consecutively included the TTEs from individuals ≥20 years old from January 1, 2017 to July 23, 2020. The inclusion and exclusion criteria for participant selection are summarized in **Supplementary Table 1**. Importantly, for multiple TTEs from a single individual, only the earliest TTE data were included for further analysis. We collected age, gender, systolic/diastolic function parameters, including LVEF, fractional shortening, E, A, E/A ratio, E/e’ ratio, septal e’, and lateral e’. Importantly, we recorded the departments that the patients were admitted to, and their primary diseases were presumed accordingly. Height and weight were not recorded in the database, and therefore, body surface area and indexed LV were not available in this study. The study was approved by the ethics committee of the First Affiliated Hospital of Nanjing Medical University (ID: 2020-SR-597). Due to the retrospective nature of the study and fully anonymized health data, the requirement for informed consent was waived [24].

### 2.3 Statistical Analysis

Characteristics of the study population were summarized according to gender. Continuous variables were presented as mean ± standard deviation (normal distribution) or median plus interquartile range (skewed distribution). Categorical variables were presented as percentages. The one-way ANOVA test (normal distribution), Kruskal-Wallis test (skewed distribution), and chi-square test (categorical variables) were used to determine statistical differences. Kolmogorov-Smirnov test was used to assess the normality. Distributions of LVEF, E/A, E/e’, septal e’, and lateral e’ were illustrated by age and gender groups using kernel density estimation with Gaussian kernels. Generalized additive models were used to assess the association between age and systolic/diastolic function parameters in males and females, and smooth trajectories of LVEF, E/A, E/e’, septal e’, and lateral e’ were calculated accordingly. The comparison of correlations was performed by using the ‘cocor’ tool [25]. The crossing point of gender-specific age trajectories of E/A ratio was considered as the point of minimal predicted difference of the generalized additive models. Correlations between variables were analyzed using the Spearman correlation coefficient. Moreover, we used multivariable regression models to reveal the impact of age and gender on LV function, and the effect of admission department and inpatient/outpatient status was accounted for the adjusted model. All statistical analysis was performed by R software version 3.6.1 (R Foundation for Statistical Computing, Vienna, Austria). *p*-value < 0.05 was considered statistically significant.

## 3.Results

### 3.1 Study Population

**Fig. 1.**
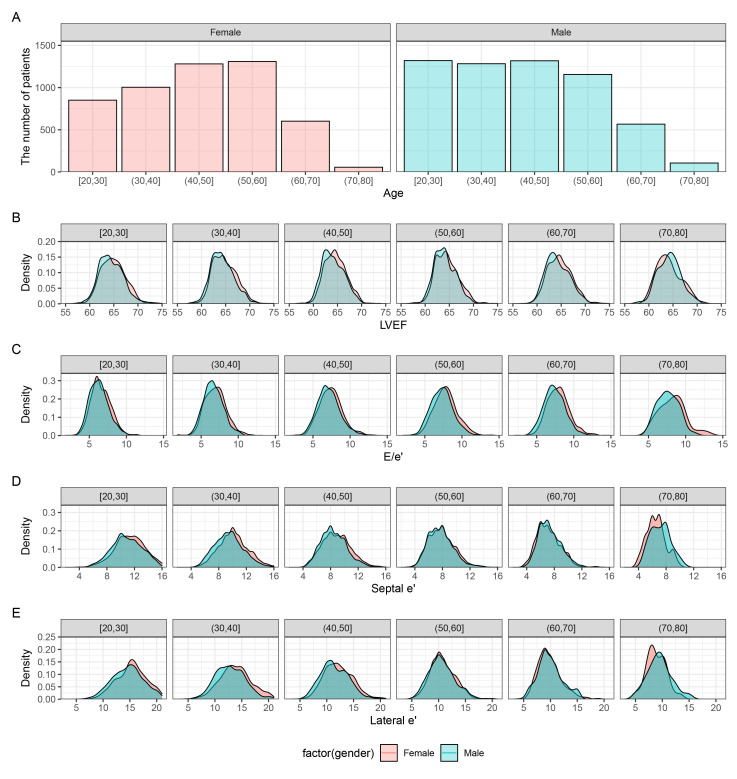
**Distribution of left ventricular systolic/diastolic function values across age in males and females**. (A) Histograms of age distribution in male and female individuals. Kernel density based on Gaussian kernels to display an overlay of female and male (B) LVEF, (C) E/e’, (D) septal e’, and (E) lateral e’. LVEF, left ventricular ejection fraction; E, early diastolic mitral inflow velocity; e’, early diastolic mitral annular tissue velocity.

A total of 10,853 individuals were enrolled in this study, including 5750 males and 5103 females with a median age of 45 years old. Population characteristics are presented in Table 1. Fig. 1A shows the number of patients for each decade of age, whereas the distributions of LVEF, E/e’, septal e’, and lateral e’ are displayed by male and female, respectively in Fig. 1B–E. Additionally, the distribution of E/A is shown in Fig. 1A. Table 2 indicates the median and interquartile range of EF, E/A, E/e’, septal e’, and lateral e’ for each age group.

**Table 1.** **Characteristics of study population**.

	Overall	Female	Male	*p*
N	10,853	5103	5750	
Age (y)	45.0 [33.0, 54.0]	47.0 [35.0, 55.0]	43.0 [31.0, 53.0]	<0.01
LVDd (mm)	46.0 [43.0, 48.0]	45.0 [42.0, 47.0]	47.0 [45.0, 49.0]	<0.01
LVDs (mm)	30.0 [28.0, 31.0]	29.0 [27.0, 30.0]	30.0 [29.0, 32.0]	<0.01
FS (%)	34.90 [34.0, 36.40]	34.90 [34.0, 36.40]	34.80 [34.0, 36.20]	<0.01
EF (%)	64.40 [62.70, 66.30]	64.40 [63.0, 66.30]	64.0 [62.40, 65.80]	<0.01
E/A	1.10 [0.8, 1.40]	1.10 [0.8, 1.4]	1.10 [0.8, 1.4]	0.969
E/e’	7.0 [6.0, 8.10]	7.30 [6.3, 8.3]	6.80 [5.9, 7.8]	<0.01
Septal e’ (cm/s)	9.0 [7.0, 10.50]	9.0 [7.0, 10.7]	9.0 [7.0, 10.3]	0.078
Lateral e’ (cm/s)	12.0 [10.0, 14.50]	12.0 [10.0, 14.9]	12.0 [10.0, 14.3]	0.06
Inpatient/outpatient	4782/6071 (44.1/55.9)	2172/2931 (42.6/57.4)	2610/3140 (45.4/54.6)	<0.01
Department of cardiology (yes/no, %)	6667/4186 (61.4/38.6)	2959/2144 (58.0/42.0)	3708/2042 (64.5/35.5)	<0.01

LVDd, left ventricular end-diastolic dimension; LVDs, left ventricular end-systolic dimension; FS, fractional shortening; EF, ejection fraction; E, early diastolic mitral inflow velocity; A, late diastolic mitral inflow velocity; e’, early diastolic mitral annular tissue velocity.

**Table 2.** **The distribution of left ventricular function parameters across age and sex**.

Age		(20, 30)	(30, 40)	(40, 50)	(50, 60)	(60, 70)	(70, 80)
Variable		Male					
	N	1320	1283	1318	1156	567	106
	EF (%)	64.20 (62.70, 66.30)	64.00 (62.70, 65.80)	64.00 (62.40, 65.80)	64.00 (62.40, 65.60)	64.00 (62.55, 66.30)	64.40 (62.47, 65.75)
	E/A	1.40 (1.20, 1.60)	1.20 (1.00, 1.40)	1.10 (0.80, 1.30)	0.90 (0.80, 1.10)	0.80 (0.70, 1.00)	0.70 (0.70, 0.90)
	E/e’	6.20 (5.40, 7.10)	6.60 (5.80, 7.50)	7.00 (6.10, 8.00)	7.30 (6.30, 8.30)	7.40 (6.40, 8.40)	7.50 (6.47, 8.62)
	Septal e’ (cm/s)	11.00 (9.50, 12.50)	9.40 (8.00, 11.00)	8.10 (7.00, 9.90)	7.75 (6.30, 9.00)	7.00 (6.00, 8.30)	7.00 (6.00, 8.00)
	Lateral e’ (cm/s)	15.00 (12.90, 16.80)	13.00 (11.00, 15.00)	11.00 (9.90, 13.20)	10.25 (9.00, 12.00)	10.00 (8.39, 11.00)	9.45 (8.00, 10.93)
	Inpatient/outpatient	386/934 (29.24/70.76)	442/841 (34.45/65.55)	648/670 (49.17/50.83)	689/467 (59.60/40.40)	378/189 (66.67/33.33)	67/39 (63.21/36.79)
	Department of cardiology (yes, %)	948 (71.82)	889 (69.29)	867 (65.78)	673 (58.22)	288 (50.79)	43 (40.57)
Variable		Female					
	N	851	1004	1281	1309	602	56
	EF (%)	64.70 (63.00, 66.40)	64.70 (63.00, 66.40)	64.40 (63.00, 66.30)	64.40 (62.70, 66.30)	64.40 (63.00, 66.30)	64.00 (62.32, 65.65)
	E/A	1.40 (1.20, 1.70)	1.30 (1.10, 1.50)	1.10 (0.90, 1.30)	0.90 (0.80, 1.10)	0.80 (0.70, 0.90)	0.70 (0.60, 0.80)
	E/e’	6.40 (5.70, 7.40)	6.90 (5.90, 7.90)	7.40 (6.40, 8.40)	7.80 (6.80, 8.80)	7.90 (6.90, 8.80)	8.25 (6.88, 9.33)
	Septal e’ (cm/s)	11.40 (10.00, 13.00)	10.00 (9.00, 11.40)	9.00 (7.40, 10.00)	8.00 (6.50, 9.00)	7.00 (6.00, 8.00)	6.60 (5.80, 7.12)
	Lateral e’ (cm/s)	15.40 (13.60, 17.30)	14.00 (12.00, 16.00)	12.00 (10.00, 14.00)	10.50 (9.00, 12.00)	9.50 (8.20, 11.00)	8.80 (7.90, 10.00)
	Inpatient/outpatient	274/577 (32.20/67.80)	338/666 (33.67/66.33)	564/717 (44.03/55.97)	643/666 (49.12/50.88)	321/281 (53.32/46.68)	32/24 (57.14/42.86)
	Department of cardiology (yes, %)	537 (63.10)	613 (61.06)	740 (57.77)	713 (54.47)	325 (59.99)	31 (55.36)

EF, ejection fraction; E, early diastolic mitral inflow velocity; A, late diastolic mitral inflow velocity; e’, early diastolic mitral annular tissue velocity.

### 3.2 Influence of Age and Gender on LVEF

**Fig. 2.**
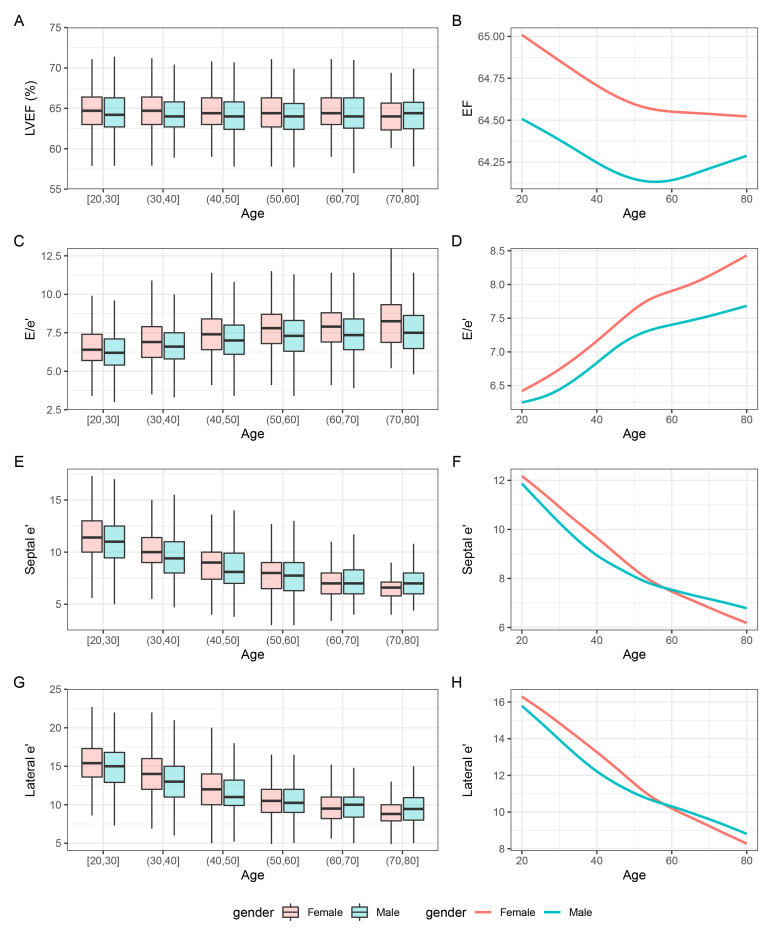
**Box plots and smooth trajectories of (A,B) LVEF, (C,D) E/e’, (E,F) septal e’, and (G,H) lateral e’ by sex and age**. LVEF, left ventricular ejection fraction; E, early diastolic mitral inflow velocity; e’, early diastolic mitral annular tissue velocity.

**Fig. 3.**
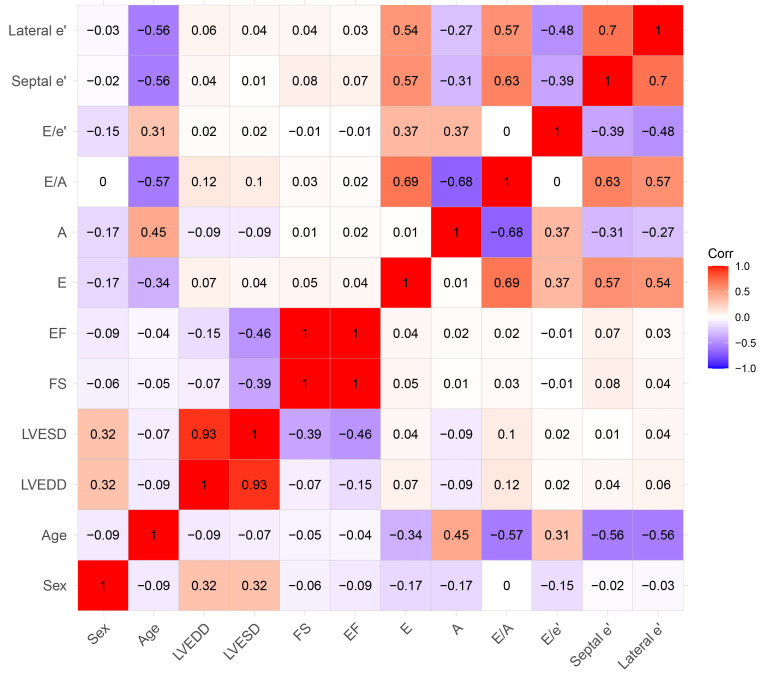
**Correlations between variables using the Spearman correlation coefficient**. FS, fractional shortening; EF, ejection fraction; E, early diastolic mitral inflow velocity; A, late diastolic mitral inflow velocity; e’, early diastolic mitral annular tissue velocity; Corr, correlation coefficient; LVDd, left ventricular end-diastolic dimension; LVDs, left ventricular end-systolic dimension.

Females showed significantly higher LVEF than males (*p* < 0.001). Fig. 2A shows the absolute values of EF for each decade of age in males and females. A significant but weak correlation between age and EF was observed (total individuals: r = –0.04, *p* < 0.001; males: r = –0.04, *p* < 0.001; females: r = –0.05, *p* < 0.001; Fig. 2B and Fig. 3). Additionally, LVEF was significantly lower in postmenopausal females (defined as >51.4 years old [26]) compared with females below premenopausal age (*p* < 0.001, **Supplementary Table 2**). Multivariable regression analysis showed that age decade (B-coefficient = –0.077, *p* < 0.001) and male gender (B-coefficient = –0.440, *p* < 0.001) were significantly associated with LVEF (Table 3).

**Table 3.** **Multivariable regression analysis of age and sex on left ventricular function**.

		Crude model		Adjusted model	
		B-coefficient	*p*	B-coefficient	*p*
EF					
	Age (decade)	–0.085	<0.001	–0.077	<0.001
	Male gender	–0.438	<0.001	–0.440	<0.001
E/e’					
	Age (decade)	0.326	<0.001	0.322	< 0.001
	Male gender	–0.394	<0.001	–0.404	< 0.001
Septal e’					
	Age (decade)	–0.994	<0.001	–0.962	<0.001
	Male gender	–0.310	<0.001	–0.290	<0.001
Lateral e’					
	Age (decade)	–1.324	<0.001	–1.293	<0.001
	Male gender	–0.492	<0.001	–0.463	<0.001
E/A					
	Age (decade)	–0.154	<0.001	–0.151	<0.001
	Male gender	–0.035	<0.001	–0.034	<0.001

Crude model: we did not adjust any covariates. Adjusted model: we adjusted for departments involved in the care of these individuals, including both inpatient outpatient departments. EF, ejection fraction; E, early diastolic mitral inflow velocity; A, late diastolic mitral inflow velocity; e’, early diastolic mitral annular tissue velocity.

### 3.3 Influence of Age and Gender on E/e’ Ratio

Females presented with a higher E/e’ ratio compared to males across all age ranges (*p* < 0.001). Fig. 2C illustrates the distribution of absolute values of E/e’ ratio in males and females. Age was significantly correlated with E/e’ both in males (r = 0.28, *p* < 0.001) and females (r = 0.31, *p* < 0.001) (Fig. 2D). Interestingly, age showed a stronger correlation in patients <51.4 years old compared with those ≥51.4 years old in both males (0.23 vs. 0.05, *p* < 0.001) and females (0.25 vs. 0.06, *p* < 0.001). Multivariable regression analysis revealed that age was a significant variable for E/e’ ratio (B-coefficient = 0.32, *p* < 0.001), as well as gender (B-coefficient = –0.404, *p* < 0.001, Table 3).

### 3.4 Influence of Age and Gender on Septal e’ and Lateral e’

Females showed similar septal e’ (*p* = 0.078) and lateral e’ (*p* = 0.06) compared with males (Fig. 2E,G). In patients <51.4 years old, females showed statistically higher septal and lateral e’ values than males (both *p* < 0.001, **Supplementary Table 3**). However, no significant gender-related difference was observed in patients ≥51.4 years old (Septal e’ *p* = 0.576, lateral e’ *p* = 0.157, **Supplementary Table 4**). Moreover, age was statistically associated with septal e’ and lateral e’ (Fig. 2F,H). For septal e’ value, the correlation coefficient was –0.59 in females and –0.54 in males, whereas the correlation coefficient between age and lateral e’ value was –0.59 in females and –0.52 in males. Interestingly, age showed a stronger impact on the septal e’ (*p* = 0.007) and lateral e’ (*p* < 0.001) of females compared with males. In the adjusted multivariable regression model, age was a significant variable for septal e’ (B-coefficient = –0.962, *p* < 0.001), as well as male gender (B-coefficient = –0.290, *p* < 0.001). Similarly, age (B-coefficient = –1.293, *p* < 0.001) and male gender (B-coefficient = –0.463, *p* < 0.001) were also important variables for lateral e’ (Table 3).

### 3.5 Influence of Age and Gender on E/A Ratio

Females showed a similar E/A ratio with males (*p* = 0.969), and the E/A ratio decreased with advancing age in both genders (**Supplementary Fig. 1**). Consistently, generalized additive models showed a significant negative association between age and E/A ratio in males (r = –0.53, *p* < 0.001) and females (r = –0.58, *p* < 0.001). The correlation between age and E/A ratio was stronger in females than males (*p* < 0.001). Interestingly, the age and gender trajectories suggested a crossing point for the E/A ratio at about 56 years of age. Multivariable regression analysis showed that age and gender were significant variables for E/A ratio (age decade: B-coefficient = –0.151, *p* < 0.001; male gender: B-coefficient = –0.034, *p* < 0.001, Table 3).

## 4. Discussion

It is increasingly clear that the hearts of males and females are not equivalent, which results in varied clinical profiles and disease outcomes [27, 28, 29]. In female patients, heart failure is usually associated with impaired diastolic function, while systolic dysfunction is a primary cause of heart failure in males [30, 31]. Moreover, some cardiovascular diseases (e.g., heart failure) are frequently underdiagnosed or diagnosed late in female patients, which might be caused by the misclassification of LV function due to inappropriate cutoff values [32, 33]. Additionally, many animal studies also suggested that male animals had a higher risk of cardiac dysfunction and/or ventricular dilation in response to stress (e.g., pressure overload) [34, 35]. Accordingly, age- and gender-specific LV function is fundamental for risk stratification and optimal health care.

Although the impact of age and gender on LV function is a hot topic with significant interest [36], it has only been assessed in small populations, which yielded conflicting results. Some studies suggested no difference in LVEF between young and the old individuals [37, 38], while others reported decreased heart function with advancing age [39, 40]. These controversial observations might be caused by different patient populations or small patient numbers. Additionally, these trials are primarily focused on European populations, and there is not enough evidence on the Asian population.

In this study, we reported the distribution of LVEF, E/A, E/e’, septal e’, and lateral e’ values obtained by TTE in a large Chinese population and revealed the impact of age and gender on LV function. To our best knowledge, this study is the first to present the lifetime trajectories of LVEF, E/A, E/e’, septal e’, and lateral e’ values from young individuals to the elderly. Our results show that females present with a higher LVEF, E/A, E/e’, septal and lateral e’ ratio than males. We observed increased E/e’ and decreased LVEF, septal e’, lateral e’, and E/A ratio with advancing age. Moreover, LVEF shows a weak correlation with age and gender, while diastolic function was shown todeteriorate more in females than males.

Aging was statistically associated with decreased LVEF, septal e’, lateral e’ and E/A ratio, with a steeper decline in females. Similarly, E/e’ increased with advancing age, and the age-specific alterations were more significant in females than males. Together with previous research [41, 42], our results highlight the necessity of age- and gender-adjusted EF, E/A, and E/e’ values. Although our research did not elucidate the mechanisms underlying the difference in LV function, several studies have provided in-depth insights into the possible mechanisms. First, myocyte hypertrophy caused by elevated aortic stiffness and afterload may be an important contributor to an age-dependent increase in LVEF [9, 43]. Recent studies further suggest that age-related increases in aortic impedance directly enhance end-systolic elastance (Ees, quantified as ESP/ESVi), particularly in healthy elderly women [44, 45]. This hemodynamic adaptation leads to characteristic structural remodeling—smaller LV cavity dimensions, increased relative wall thickness (RWT), and hyperdynamic systolic function—which collectively contribute to the observed supra-normal LVEF in this population. An autopsy study has demonstrated an age-related progressive myocyte loss in males but not in females [46], suggesting sex-specific pathways of cardiac aging. Moreover, changes in hormone (e.g., estrogen, testosterone, and insulin-like growth factor 1) status are also a significant contributor to the age- and gender-related differences in LV function [42]. Menopause has been demonstrated to accelerate vascular stiffening [47, 48, 49, 50], which causes unfavorable deteriorating cardiovascular status over 6–10 years [48, 50]. Decreased testosterone levels in elderly male individuals will result in lower cardiac sympathetic nerve activity [51]. Insulin-like growth factor 1 has also been demonstrated to be associated with the inotropy of LV [52]. Accordingly, it is speculated that the alterations in hormone levels are responsible for the age- and gender-related differences. Furthermore, age has a more pronounced impact on the heart function of females. However, the causes remain vague, and it is still unclear whether these alterations in elderly females would have a beneficial or detrimental influence on cardiovascular disease-related morbidity and mortality.

Several limitations of this study should be pointed out. The cohort comprised individuals referred for TTE with “normal” results, not random healthy samples, introducing selection bias: rigorous exclusions (e.g., structural abnormalities) may over-represent healthier individuals, while residual pathologies (e.g., undiagnosed myocardial infarction) and comorbidities (e.g., hypertension, diabetes) could confound age/gender effects. The retrospective design of the study precluded the collection of critical variables (e.g., body surface area for indexing LV parameters), limiting the amount of adjustment for residual confounders. Findings are specific to TTE and a single-center setting, potentially reducing the generalizability of the findings to other imaging modalities (CT/MRI) or institutions. Additionally, incomplete diastolic assessment (missing left atrial volume and tricuspid regurgitation velocity) weakened the diastolic function evaluation. While the inclusion of comorbidities aimed to reflect real-world populations, these biases must evoke caution in interpreting the observed age- and gender-related LV function differences.

## 5. Conclusion

This study provided the distribution of LV systolic/diastolic function across age decades in males and females. We observed a strong correlation of age with E/A and E/e’ but a weak correlation with LVEF, and these alterations were more pronounced in females. Our study highlighted the necessity of age- and gender-specific criteria in clinical decision making and emphasized the focus on ventricular function for patients with normal cardiac structure.

## Availability of Data and Materials

All data included in this study are available upon request by contact with the corresponding author.

## Supplementary Material

Supplementary material associated with this article can be found, in the online version, at https://doi.org/10.31083/RCM38779.
