# Supplementary material for: The Impact of Age and Sex on Left Ventricular Function Based on Transthoracic Echocardiograms
Source: Rev Cardiovasc Med. 2025 Jun 27;26(6):38779. doi: 10.31083/RCM38779 (PMC12230827; doi:10.31083/RCM38779)
Supplement: Supplementary file 1 [file 2153-8174-26-6-38779-s1.zip › Supplementary Material.docx]

**Supplementary material**

**Supplementary Table 1. Inclusion and exclusion criteria**

| **Category** | **Criteria** | | |
| --- | --- | --- | --- |
| **Inclusion** | Unit (mm) | Min | Max |
|  | -left atrium diameter | 27 | 37 |
|  | -left ventricular end-diastolic diameter | 35 | 55 |
|  | -left ventricular end-systolic diameter | 23 | 35 |
|  | -interventricular septum | 8 | 11 |
|  | -right atrial diameter |  | 40 |
|  | -right atrial end-diastolic diameter |  | 40 |
| **Exclusion** | -atrial fibrillation | | |
|  | -hypertensive heart disease | | |
|  | -segmental wall motion abnormalities | | |
|  | -congenital heart disease | | |
|  | -valvular heart disease | | |
|  | -cardiomyopathy | | |
|  | -septal thickening | | |
|  | -pericardial effusion | | |
|  | -mirror-image dextrocardia | | |
|  | -heart surgery | | |

**Supplementary Table 2. Comparison of premenopausal (≤51.4) and postmenopausal women (＞51.4).**

|  | **[ALL] N=8203** | **Above N=2601** | **Equal or below N=5602** | **p.overall** |
| --- | --- | --- | --- | --- |
| **EF** | 64.7 [63.0;66.3] | 64.4 [63.0;66.3] | 64.7 [63.0;66.4] | <0.001 |
| **E/e’** | 7.2 [6.2;8.3] | 7.8 [6.8;8.8] | 6.9 [6.0;8.0] | <0.001 |
| **Septal e’** | 9.0 [7.3;11.0] | 7.4 [6.0;9.0] | 10.0 [8.2;12.0] | <0.001 |
| **Lateral e’** | 12.4 [10.0;15.0] | 10.0 [9.0;12.0] | 13.9 [11.6;16.0] | <0.001 |
| **E/A** | 1.2 [0.9;1.4] | 0.8 [0.7;1.1] | 1.3 [1.1;1.5] | <0.001 |

**Supplementary Table 3. Comparison of male and female (≤51.4).**

|  | **[ALL] N=11621** | **Female N=5602** | **Male N=6019** | **p.overall** |
| --- | --- | --- | --- | --- |
| **EF** | 64.4 [62.7;66.3] | 64.7 [63.0;66.4] | 64.0 [62.4;65.9] | <0.001 |
| **E/e’** | 6.7 [5.8;7.8] | 6.9 [6.0;8.0] | 6.6 [5.7;7.6] | <0.001 |
| **Septal e’** | 10.0 [8.0;11.3] | 10.0 [8.2;12.0] | 9.4 [8.0;11.0] | <0.001 |
| **Lateral e’** | 13.0 [11.0;15.2] | 13.9 [11.6;16.0] | 13.0 [10.9;15.0] | <0.001 |
| **E/A** | 1.2 [1.0;1.5] | 1.3 [1.1;1.5] | 1.2 [0.9;1.4] | <0.001 |

**Supplementary Table 4. Comparison of male and female (＞51.4).**

|  | **[ALL] N=5189** | **Female N=2601** | **Male N=2588** | **p.overall** |
| --- | --- | --- | --- | --- |
| **EF** | 64.0 [62.7;65.9] | 64.4 [63.0;66.3] | 64.0 [62.4;65.8] | <0.001 |
| **E/e’** | 7.5 [6.5;8.5] | 7.8 [6.8;8.8] | 7.3 [6.3;8.3] | <0.001 |
| **Septal e’** | 7.3 [6.0;9.0] | 7.4 [6.0;9.0] | 7.3 [6.1;8.8] | 0.576 |
| **Lateral e’** | 10.0 [9.0;12.0] | 10.0 [9.0;12.0] | 10.0 [9.0;12.0] | 0.157 |
| **E/A** | 0.8 [0.7;1.1] | 0.8 [0.7;1.1] | 0.8 [0.7;1.1] | 0.298 |

**
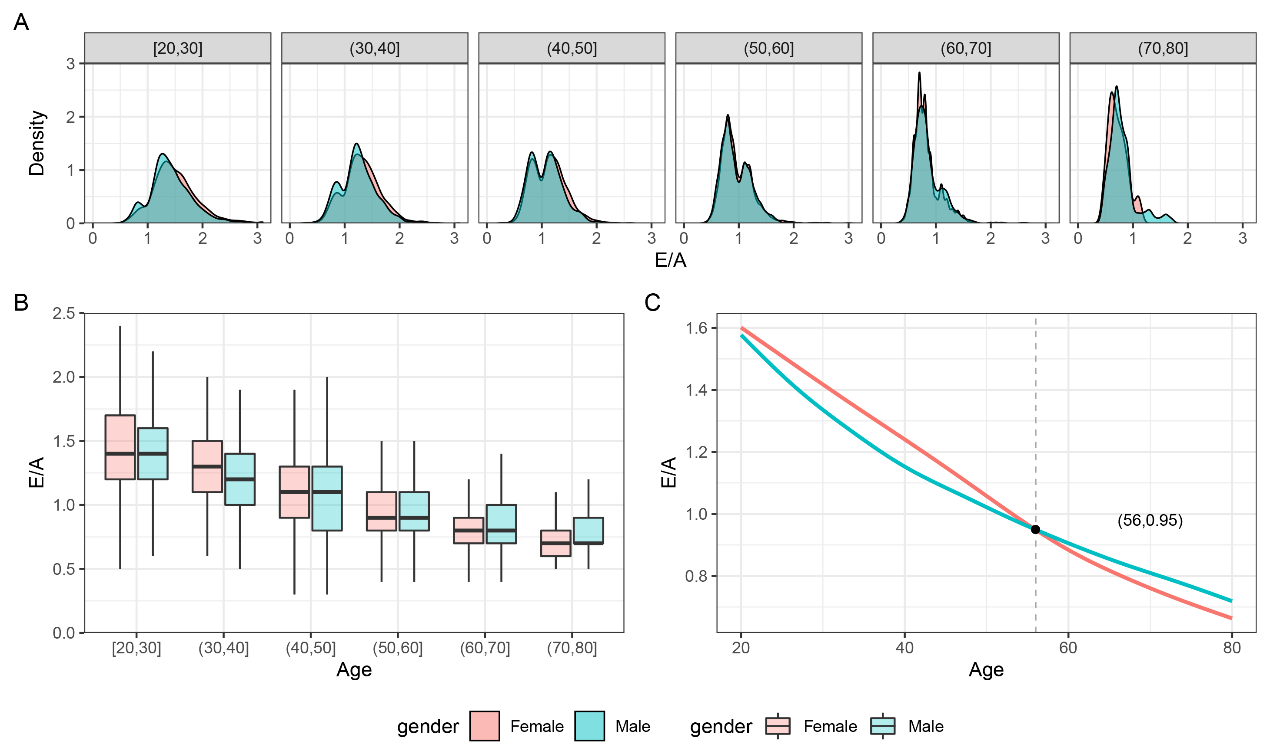
**

**Supplementary Fig. 1**. (A) Distribution of E/A across age in males and females. (B) Box plots of E/A ratio by sex and age. (C) Smooth trajectories of E/A ratio. E: early diastolic mitral inflow velocity; A: late diastolic mitral inflow velocity.
